# A longitudinal mixed methods social network analysis to evaluate a peer-led housing program for older men returning from incarceration: Study protocol & pre-implementation results

**DOI:** 10.1186/s40352-025-00362-4

**Published:** 2025-08-16

**Authors:** Brandy F. Henry, Derek A. Kreager, Joy Gray, Kristina Brant, Gary Zajac, Divine Lipscomb, Sarah Brothers, David R. Schaefer, Nicolette Bardele, Andrea Hazelwood

**Affiliations:** 1 Pennsylvania State University, University Park, PA USA; 2 University of California Irvine, Irvine, CA USA

**Keywords:** Program evaluation, Reentry, Social networks, Housing, Implementation science, Incarceration, Mental health, Substance use, Mixed methods, Peer support

## Abstract

**Background:**

We describe a longitudinal mixed methods program evaluation protocol for a novel peer-led housing program for older men transitioning from prison to the community after completing long sentences of incarceration. The program departs from traditional community corrections models by providing peer-run housing designed to build and enhance peer and community social ties. This previously untested program relies on the principles of network alteration and provides a case study for examining interpersonal mechanisms underlying behavioral health and justice related outcomes.

**Methods:**

We use mixed methods and longitudinal social network analysis to evaluate the program, while also applying implementation science to document program development. We focus our evaluation on key health and social outcomes, including mental health, substance use, stress, health risk behaviors, well-being, financial security, housing, and recidivism. With longitudinal surveys, we collect (1) dynamic network data of resident and staff relationships and (2) behavioral health/social data of participants. We also administer longitudinal resident and staff interviews. Resident interviews focus on interpersonal relationships and reentry experiences, while staff interviews describe program implementation. We apply longitudinal statistical models to complete (i.e., sociocentric) network data within the house to examine how dynamic network properties connect to changes in residents’ health, behavioral, and social outcomes. We integrate longitudinal survey, individual-level (i.e., egocentric) network, and qualitative data to understand how the program works. To evaluate program impacts for long-term health and social outcomes, we use an untreated matched sample to compare 6- and 12-months post-prison release outcomes using administrative data related to rearrest/reincarceration and behavioral health.

**Pre-implementation results:**

We use a logic model to present and organize pre-implementation results from interviews with program staff and peer mentors. Our results describe program design and intended goals, while highlighting how the program is rooted in principles of peer support, trauma-informed care, and restorative justice to address unique stressors of incarceration to foster responsibility and facilitate reintegration.

**Discussion:**

Community program evaluation research allows us to document real-world contextual factors that may drive intervention effectiveness. Results of the mixed methods evaluation will provide a comprehensive understanding of one network-based program’s ability to support health and social outcomes of older, previously incarcerated men. Results may inform future reentry services.

## Background

Close to 95% of incarcerated individuals in the United States (US) will experience reentry, with approximately 400,000 individuals entering parole from state and federal prisons every year (Kaeble, 2021; Muhlhausen, 2018). Due to longer sentences associated with increasingly punitive sanctioning, larger proportions of incarcerated individuals are spending much of their adult lives behind bars (Kazemian & Travis, 2015). Resultingly, the average age of the incarcerated population has increased substantially over time (Carson & Sabol, 2016; Porter et al., 2016), leading to more incarcerated individuals exiting confinement in mid-to-late life. Older, long-term incarcerated individuals are particularly vulnerable to health and social risks. Exiting confinement is a demanding and stressful life course transition for anyone (Western, 2018), but it is particularly difficult for this population, who are more likely to face health challenges and to have lost crucial social ties. Specific needs of this growing population are largely unmet within existing correctional and parole systems. Current parole policies are built on models of supervision and deterrence and are not well-equipped to deal with older, previously incarcerated individuals lacking the human and social capital to restart their post-prison lives (Lares & Montgomery, 2020; Latham-Mintus et al., 2023 ; Wyse, 2018). Historically, programs for this population have also not been community-based or peer-driven.

Health is intertwined with social relations across the life course, and this connection is stronger for those exiting long periods of incarceration. Health typically declines later in life, when life course transitions common to older adulthood (e.g., retirement, widowhood, dispersed children) may weaken or realign social ties (Alwin et al., 2018; Cornwell & Qu, 2024 ; Weiss et al., 2022). This normative weakening and restructuring of social networks are likely further exacerbated by long incarcerations. Limited knowledge in these areas supports the need for basic observational research focused on the network mechanisms underlying behavior change and health among previously incarcerated older adults, with the intent to inform future network and health interventions for this population. While some initiatives across the US have been designed to meet the needs of older, previously long-term incarcerated individuals before and after reentry (O’Hanlon & Broome, 2022), none have been rigorously evaluated.

The most pressing requirement for reentry success is stable, safe, and affordable housing (Western et al., 2015), a need particularly salient for older, long-term incarcerated individuals who lack network ties in the community. Beyond housing, common reentry considerations for individuals after long incarcerations include: (1) accelerated physical/mental aging and chronic health challenges resulting from the stress and conditions of incarceration, (2) the need to secure employment or income that matches their skillset and needs, (3) unfamiliarity with changes in technology, and (4) social isolation resulting from the decay of social ties while incarcerated (Colibaba et al., 2021; O’Hanlon & Broome, 2022). Given the tendency for community and family ties to erode during prolonged incarceration, older, previously incarcerated individuals are in great need of both housing assistance and social support to cope with daily reentry challenges in unfamiliar environments (Lares & Montgomery, 2020; Wyse, 2018).

The existing reentry program landscape is largely designed and delivered by correctional agencies. Services typically focus on monitoring compliance with court orders and skills training, often prohibiting interactions with other reentrants, thereby eliminating opportunities for social network building with peers. These programs also have little input from the people they aim to serve, and are infrequently based in the community, which is where reentry actually occurs. Evaluations of existing programs have produced mixed results, with even successful programs having very small effect sizes, and rarely being replicated (Petrich et al., 2022). Previous research has also struggled to holistically measure reentry success, focusing primarily on recidivism (National Academies of Sciences, Engineering, and Medicine, 2022).

Experiences of past stress and trauma are also overrepresented among incarcerated populations (Morrison et al., 2019; Wolff et al., 2014), and past stresses are exacerbated by the experience of incarceration itself. The stress imposed by incarceration often leaves people returning to their communities with psychological symptoms similar to post-traumatic stress disorder, recently described as post-incarceration syndrome (Liem & Kunst, 2014). To address this, the Substance Abuse and Mental Health Services Administration (SAMHSA) promotes the use of trauma-informed approaches for justice-involved populations. This approach realizes the widespread impact of trauma on recovery, recognizes signs of trauma in individuals and communities, and responds to trauma with knowledge and practices that resist re-traumatization, including gender-tailored programming (Substance Abuse and Mental Health Services Administration, 2014). Evidence shows that trauma-informed services can help current and formerly incarcerated individuals heal from their own victimization (Petrillo, 2021), reduce symptoms of depression and aggression, increase hope and optimism (Marrow et al., 2013), and promote positive relationships while reducing criminogenic risk factors (Skinner-Osei et al., 2019).

Alongside the movement to promote trauma-informed services, there has been a movement to promote peer-services within reentry programs, incorporate broader measures of successful reentry, and apply more restorative approaches to justice which center rehabilitation and reconciliation through the “5 R’s:” relationship, respect, responsibility, repair, and reintegration (Pavlacic et al., 2022). Early research on peer services reports that formerly incarcerated individuals participating in peer-run case management services identify supportive and non-judgmental peers who treat them with dignity, respect, and autonomy as essential to a positive reentry experience (McLuhan et al., 2023; O’Hanlon & Broome, 2022). Community-based peer housing programs also allow formerly incarcerated residents to build relationships, develop interpersonal skills, and share connections to community resources with one another (Colibaba et al., 2021). Moreover, giving back to peers and the community (such as transitioning to the role of peer mentor) provides “redemption scripts” that help individuals move beyond their criminal pasts (Maruna, 2001).

The program we study departs from traditional approaches and leverages the peer-centered movement to develop a new model for reentry. Our research approach also responds to calls for broader measurements of success and deviates from historical approaches by partnering with the community-based program to document and holistically measure the peer-driven community-based Cumberland House program.

## The Cumberland House Reentry Program

Here we describe the Cumberland House Program design as initially implemented. The Cumberland House program is part of a larger non-profit organization called Tomorrow’s Neighbors which was initially funded through an American Rescue Plan Act (ARPA) grant with buy-in from local and state stakeholders. Tomorrow’s Neighbors provides peer support for formerly incarcerated men and operates the Cumberland House as a separate project. The founder and director of Tomorrow’s Neighbors is also the founder and director of the Cumberland House.

The house is located in a residential neighborhood and can serve up to 13 men in single, double, and triple occupancy rooms. In addition to the guest rooms, there is also a staff bedroom. Residents, referred to by the program and hereafter as house “guests,” share a communal bathroom, kitchen, office, and common space. The common space is also used to host community building activities for guests, mentors, and staff. Peers connected to the related non-profit are expected to serve as mentors to house guests, and as house guests move out of the house after 3–6 months, they are expected to transition into the mentor role. House guests pay an affordable rate for a furnished room. Affordability is determined through an individualized assessment by the program director and staff where the specific economic situation of each guest is considered in tandem with house resources.

The initial program idea came from the founder and current program director. The program director is recently navigating reentry after long-term incarceration (10 + years). Prior to founding Tomorrow’s Neighbors, he was primarily a service recipient, not a service provider. The program director spent the majority of his adult life incarcerated and has little formal training in human services management or delivery. As such, he leverages his and his peers’ lived experience to design the program through a grassroots effort which is emergent and responsive to the population’s needs. To develop the Cumberland House, the program director solicited input from other older, long-term incarcerated men. These men eventually became the initial program staff and mentor team. The program aims to serve this same population through a peer-driven, community-based housing model.

The novelty of the program lies in its commitment to establishing a peer-led system of community-based support for older, previously incarcerated men as a key mechanism for facilitating reentry success. Hence, social support comes from peer staff, peer mentors, and other residents, all of whom are expected to be formerly incarcerated. This is groundbreaking in that most men are released from prison to parole, and associations between individuals on parole are typically prohibited under the assumption that they are criminogenic. This assumption comes from research citing that peer influence is a risk factor for recidivism. However, much of that research was based on younger people and gang influences. Recent research reveals that peer support reduces odds of reincarceration (Mowen & Boman, 2019). Evaluating the Cumberland House allows us an opportunity to add to this research and measure success beyond recidivism.

The peer-led model of the Cumberland House builds relationships that would normally be deemed parole violations sanctionable by community corrections authorities. Rebuking this corrections norm, the program sees such peer support as vital for the reentry success of older, previously incarcerated men. The residents in the program have received permission from parole to allow for the cultivation of relationships between residents, mentors, and staff who are formerly incarcerated and while under parole supervision. The goal is for peer relationships to culminate in a community offering support both during and after residents’ time in the house.

The focus on providing housing within a peer designed framework aligns with “housing-first” models (Padgett et al., 2016). However, the Cumberland House does not follow any existing housing models. Initial program goals were to provide stable housing and peer support to older, previously incarcerated men exiting incarceration after long prison sentences. The primary short-term goals were for participants to secure long-term housing, employment, independent living skills, behavioral health treatment (as needed), and to build supportive networks, such that they can live independently after approximately 3–6 months living in the house. The program targets men leaving prison after serving sentences of 10 + years, although there is no minimum required length of sentence to be eligible for the program. Exclusion criteria include having a severe mental health or substance use disorder or history of arson or sexual offense, the latter of which are prohibited due to insurance and zoning regulations.

The program director and staff individually assess program eligibility and fit during interviews with applicants. The process of determining fit is informal and filtered through the lived experience of the staff and their knowledge of the current house dynamics. Program fit is established through evidence of commitment to personal growth and shared values of the house. Given that most men are on parole which mandates drug testing, there is also an expectation of sobriety within the house. An example of poor fit might be someone who shows no evidence of engaging in personal growth during incarceration, and/or has no interest in developing peer relationships with other house guests.

The subjectivity of the program eligibility process is viewed by the program as a strength and stems from an underlying value of the Cumberland House of the philosophy of “nothing about us without us,” originally coined by the disability justice movement (Charlton, 1998). This principle centers lived experience and rejects carceral norms as the foundation of the program. As such, the program embraces oral communication and is not formally documented through manuals, client charts, or intake forms. The program rejects manualization and strives to be dynamic, adapting to the individualized needs of house guests and the lived experiences of house mentors. Therefore, one of the goals of our research is to formally describe the program so that the intervention can be tested and scaled. Part of our research protocol is also to document program implementation and change as it occurs. This process is in line with the emergent design approach which engages in program evaluation using a loose participatory framework to document program development and change alongside evaluation (Christie et al., 2005).

The shift away from manualized and bureaucratic processes is in line with many practitioner and community member perspectives that such approaches are not sufficiently adaptable or responsive to the skills of practitioners or the needs of clients. Further, a systematic review found that while manual-based treatment facilitates research, it is not clinically superior to nonmanualized clinical practice (Truijens, Zühlke‐van Hulzen, & Vanheule, 2019).

### Methods and design

#### Study aims

Our study breaks new ground in documenting and measuring the dynamic and adaptable Cumberland House program. In order to achieve this, a novel element of our study is our mixed-methods approach, which applies social network analysis, ethnography, in-depth interviews, surveys, and implementation science to longitudinally evaluate the Cumberland House program, while also documenting the complex reintegration process for older men leaving prison. Including holistic measures on the overall experience of reentry allows us to document mechanisms of reentry success within and outside of the Cumberland House. We focus our evaluation on key health and social outcomes, including mental health, substance use, trauma, health risk behaviors, overall well-being, financial security, housing, and recidivism. Our four aims include:

1. Track relationship development and the provision of peer social support among older, long-term incarcerated men.
2. Connect the program and social network processes to long-term individual outcomes.
3. Explore program mechanisms with mixed methods social networks analysis (MMSNA).
4. Describe program implementation to support future intervention design and scalability.

### Sampling

Data collection began in the summer of 2023 and is planned to continue through 2027. All house guests (N = up to 13 at one time), peer mentors (*N* = 5 at program start-up), and peer staff (*N* = 2 at program start-up) are eligible research participants while residing/working at the house and three months after exiting the house. With guest stays of approximately 3–6 months, we expect a total of 80–100 eligible guest participants. Participants are provided with incentives for research participation (local supermarket or Amazon gift cards). All participants give informed consent. Our study is approved by the Pennslyvania State University Institutional Review Board.

### House guest data

During residence in the house (approximately the first 6 months of reentry), we gather intensive data on social network development and critical outcomes using (1) monthly surveys to assess each guest’s ties inside the house and health/reentry outcomes, and (2) interviews at three time points; within one week of a guest’s arrival and in months three and six. These two components emphasize different aspects of guests’ networks. The monthly surveys track the status of ties inside the house (i.e., to fellow guests, mentors, and staff) by asking residents to select who they have various types of relationships with from a roster of current house affiliates. This form of “sociocentric” network data (i.e., ties within a bounded population) is complemented by “egocentric” network data collected during interviews using Network Canvas, an open-source software tool for collecting and visualizing social network data (Janulis et al., 2023).

Egocentric data offers insight into a wider swath of relationships, which could include people inside or outside the house and who may provide a wider range of support. Collecting this type of data is time-intensive, but is more readily paired with open-ended reflections on the people in one’s network, their role during reentry, and subjective changes in health and other reentry experiences. After each guest has moved out of the house, we also follow up with three monthly surveys and one final interview three months later. While baseline surveys take approximately one hour to complete, follow-up surveys are shorter, at approximately 30 min each. Surveys are completed either independently on personal devices, or via Computer-Assisted Personal Interview (CAPI), where researchers read questions to participants and record responses on project computers, or individually on personal laptops. Interviews take one to two hours to complete. Given the sensitive nature of some of our questions, there is potential for participants to experience stress during data collection. Therefore, we have support available through our research team to assist in risk assessment, brief support, and referral as needed.

#### Baseline demographic survey data

All house guest participants provide self-reported data for age, race, religious affiliation and engagement, highest level of education prior to incarceration, educational or vocational training received in prison, marital and/or relationship status and quality, number of children (biological, step, or adopted), length of incarceration, probation or parole status, employment prior to incarceration, and employment-seeking interests since incarceration.

***Health & social outcome survey data***: Monthly guest surveys measure health and social outcomes across multiple sub-categories: physical functioning, fatigue, sleep quality, health risk behaviors (i.e., sexual health behaviors), worthiness/perceived hope/purpose, substance use (Justice Community Opioid Innovation Network core measures, and The Tobacco, Alcohol, Prescription medications, and other Substance (TAPS) Tool [Justice Community Opioid Innovation Network Coordination and Translation Center, 2023; Wu et al., 2016]), depression (Patient Health Questionnaire (PHQ-9) [Kroenke et al., 1999]), anxiety, despair, (World Health Organization Quality of Life (WHOQOL-BREF), [World Health Organization Quality of Life Group, 1998]), meaning in life (Salsman et al., 2020), and post-incarceration syndrome (Liem & Kunst, 2014). Measures are validated and reliable. We additionally include validated measures of social disconnectedness, stigma (Krieger et al., 2005), trauma/stress (Brief Trauma Screening Questionnaire (Brewin et al., 2002); Stressful Life Events Screening Questionnaire, Revised (Goodman et al., 2013), and perceived isolation (Cornwell & Waite, 2009). We also measure reincarceration.

#### Social network survey data

In-house networks are measured using a roster of current house guests, staff, and mentors. This roster is updated regularly as guests move in and out of the house, and as mentors enter or leave the program. During each survey, we ask respondents who in the past month (1) they got along with most, (2) provided them help/support, (3) is a leader, and (4) has been the most demanding or difficult. These nominations provide complete (i.e., sociocentric) data within the bounded contexts of the house on four relationship types each month. Open-ended follow-up questions ask respondents to elaborate on the nature of specific relations. During the baseline survey, we ask how well they knew each nominated alter prior to joining the program, and we phrase the remaining network items prospectively (e.g., who do you think will be most supportive, and why). Once guests move out of the house, we shift to a name-generator format which is a survey question that asks which current or former program affiliates they have contacted in the past month, with follow-up questions asking about the nature of support exchanged in each relationship.

#### Social network and reentry experience qualitative data

More comprehensive data on guests’ networks are obtained through semi-structured interviews. Within the interviews, we use Network Canvas to solicit egocentric network data on relationships with people whether they are inside or outside the house. Examples of relationships outside the house include family members, romantic partners, and parole agents. At the baseline interview, two name generators ask guests to list (1) people to whom they have felt close, from whom they have received support, or with whom they have discussed important matters and (2) people they have encountered most regularly, even if they are not close to them. Follow-up name interpreter questions elicit demographic information for each person mentioned. Within Network Canvas, guests then visualize their egocentric network by placing each person listed onto concentric circles indicating their level of closeness and drawing ties among all alters. We then probe for qualitative information about the network, including support received and provided, particularly difficult and fulfilling relationships, relationships the guest hopes to strengthen, and potentially negative influences. We also probe for further information about guests’ network structures. A final name generator asks guests to list anyone with whom they would like to connect or reconnect in the next few months in order to collect data on anticipated ties.

In follow-up interviews, we collect data on network change by revisiting the network generated during the prior interview, focusing on both dissolved and nascent ties. We ask guests about any changes in the relationships mentioned, including whether any strengthened or worsened and whether any relationships ended entirely, with follow-up questions probing why (Fischer, 2018). We also use the same name generators mentioned above to add any new ties to the network, and we again probe for similar themes of support received and provided and other qualitative dimensions of guests’ relationships and network structures. To gain insight into whether anticipated ties ultimately develop, we revisit the prior list of people guests mentioned with whom they would like to connect or reconnect and probe for whether these ties have developed (and why or why not).

Alongside data on guests’ social network development, other qualitative data on guests’ experiences in the house and broader reentry experiences are obtained through the interviews. Interviews cover topics including (1) barriers and benefits of the reentry program in relation to reaching personal milestones and goals, (2) physical/mental health, stress/coping mechanisms, drug/alcohol use, and access to medical care, (3) housing, education, employment, and participation in community and religious groups/organizations, (4) expectations for the future, and (5) emotional and lived experiences of reentry. We conduct interviews within one week of a guest’s arrival, in month three, and in month six. We also interview guests three months after they have left the house, asking guests additional questions about the house’s impact on them and their post-house reentry experiences. The first interview always occurs in a private location in the house, while subsequent interviews may occur in the house or by phone or Zoom. Interviews are recorded and transcribed for analysis.

### Peer mentor and staff data

We survey peer mentors and staff approximately every two months, focusing on their relationships with house guests and perceptions of guest progress and house social structure. Initial surveys also ask about their own incarceration experiences. Network data are gathered by providing mentors and staff with a roster of current house guests and asking them to identify whom they have mentored, who are leaders in the house, and an open-ended question regarding who has been successful or struggled between surveys. This question is intentionally left vague to allow the mentors to describe their perception of success or struggle. Additional, follow-up open-ended questions inquire about what makes named guest(s) a leader and their impressions of why named guests are succeeding or struggling.

We also conduct two open-ended, in-depth interviews with each mentor and staff member—one at baseline or when they join the program, and one at the end of study year 2 to understand and assess program implementation. Staff members are also interviewed every 2 months on key elements of program development and barriers/facilitators to its implementation. These interviews take place in the house or on Zoom and follow similar procedures as guest interviews.

From mentors and staff, we also collect demographic information in their baseline survey on age, race, religious affiliation and engagement, probation or parole status, highest level of education prior to incarceration, educational or vocational training received while incarcerated, and related volunteer experience. Regarding mental health status, we ask if mentors/staff are in recovery from a substance use disorder and if they have a prior or current mental health disorder.

### Ethnographic data

During study years 1–2, we also conduct a longitudinal ethnography of the house, with an emphasis on participants’ behaviors and interactions. Researchers spend time with participants in common areas of the house, and attend group activities and events in the house and surrounding areas. Each study participant decides how much interaction to have with the researchers in ethnographic settings. Researchers interact with those participants who have provided informed consent through free-flowing conversations, thereby prompting spontaneous statements regarding social networks and daily life activities.

This participant observation data collection strategy permits cross-checking and triangulation of data. Researchers record fieldwork jottings that are typed up into full field notes following observations. Some ethnographic observations may also be audio-recorded (ensuring that all present have consented to the ethnographic study). Notes are structured around the specific aims of the project. As notes are completed, we flag important themes to consider for subsequent coding/analysis and discuss emergent themes to be considered as additional survey items.

### Administrative data

At the conclusion of primary data collection, secondary administrative records will be obtained for participants and a matched sample to track long-term criminal justice and health outcomes (6 months and 1 year after prison release). We will acquire rearrest and reincarceration data and Medicare/Medicaid service records through a secure administrative data storage center. With participant consent, we will access person-level data files which will be merged with other survey data. We will create a comparison group of previously incarcerated individuals matched to our study sample by age, sentence length, offense type, race, and committing county. To the extent possible, we will also match on variables which would overlap with “program fit,” for example, participation in education, or other programming during incarceration, and absence of a disciplinary record. We will compare rearrest, reincarceration, and health outcomes for this group to the study sample to provide another measure of program impact.

### Implementation science data

We assess program acceptability and feasibility through mentor/staff surveys, using the validated Acceptability of Intervention Measure (AIM) and Feasibility of Intervention Measure (FIM). Both measures include 4 items and use a 5-point scale, with higher scores indicating higher acceptability (Weiner et al., 2017). Both mentors/staff and guests will provide survey data to score intervention key elements. We will also include open-ended items for mentors/staff aimed at their current understanding of the program, their mentoring strategy, and experiences with guests. In-depth interviews are also conducted with staff approximately every two months to discuss program operation, and implementation, including barriers and facilitators.

### Data analysis plan

***Aim 1: Track relationship development***: To understand individual network development, we will track changes of in-house and out-of-house network measures over time. Using the sociocentric data on in-house ties, we will measure changes in guest position (e.g., outdegree, indegree, mutuality, triadic closure, multiplexity) (Wasserman & Faust, 1994) for each type of relation over time. Based on the program model, we expect that guests who develop stronger, more central positions in house support and leadership networks will experience more successful reentry outcomes, as evidenced through measures of employment, long-term housing, behavioral health and well-being. As appropriate, we will also calculate egocentric network measures using the interview data that provides information on out-of-house ties (e.g., outdegree, multiplexity). We expect that extensive relationships with program affiliates will emerge early and persist across the study period, while community-based relationships and involvement emerge later in reentry. We will track which mentors were former guests and include a control for this in analyses that include measures of mentors.

*Tie origins*. Using the sociocentric data on in-house ties, we will investigate what leads some guests to be seen as leaders, helpful, or difficult. We will estimate Exponential Random Graph Models (ERGMs) (Robins et al., 2007) that predict being nominated for each of these relation types based on factors that carry status in prison, such as age, tenure in program, or conviction background (Kreager et al., 2017). We will pool data across months (Hanneke et al., 2010), using a block diagonal approach that provides an efficient way to avoid scaling bias (Duxbury & Wertsching, 2023). Using the egocentric data, which includes ties outside the house, we will evaluate patterns in relationship origins by examining how they met (i.e., through work, an informal group, via a program affiliate, etc.) and other factors to predict which ties persist, using multilevel models to account for nesting of ties within guests (Perry et al., 2018).

*Program structure*. We will use the sociocentric data (which are monthly panels) to calculate several network-level statistics that shed light on how the house’s informal structure shifts across time (e.g., centralization, clustering, homophily). This will offer insight into questions such as how the presence of clear leaders changed or whether the house was more or less cohesive versus cliquish across time. We expect, for instance, that the distribution of outdegree on support given will be unequal, but it should not be so unequal that only 1–2 people are providing the bulk of support, which would threaten sustainability. We will estimate pooled ERGMs to investigate the factors that drive the different relations constituting house structure, such as homophily on background, mutuality, and transitivity, which have been shown to foster friendships in prison (Schaefer et al., 2017).

***Aim 2: Connecting program participation and network processes to individual outcomes***: We will use the measures of individual network position calculated for Aim 1 to predict participant well-being and other reentry outcomes. Using multilevel models to account for repeated measures, we will estimate how changes in network integration explain key indicators of reentry success (e.g., securing housing and employment, mental health, accessing services, etc.). We will use pooled ERGMs to test how in-house network properties (e.g., indegree, homophily) are associated with these outcomes, which will help to control for network confounds related to health behaviors (Haynie et al., 2018). We will also estimate Stochastic Actor-Oriented Models (SAOM) (Snijders et al., 2010) to evaluate whether any clustering on outcomes is due to network position, peer influence, or selection.

Lastly, to estimate program effects, we will assess program participant individual level change over time on health and social measures collected through guest surveys. We will also compare program participants to a matched sample on 6-month and 1-year administrative data for rearrest, reincarceration, and health outcomes. These key programmatic outcomes will be compared between the two groups using t-tests, with effect sizes calculated using the standardized difference between group means and pooled sample standard deviations (Sullivan & Feinn, 2012).

***Aim 3: Exploring network mechanisms and program heterogeneity***: Guests enter the program with different backgrounds, attitudes, and identities that shape both their program experiences and health trajectories. We will follow a mixed-methods social network approach by complementing the survey network data and analyses of Aims 1 and 2 with narrative and observational data to understand how the network-based program works, consider how program experience varies across individuals, and identify challenges to the provision of social support and relationship formation processes. Longitudinal interviews both during and after guests’ time in the house will ask respondents to reflect on their networks, with prompts that cover network-building strategies they used, challenges they encountered, and where they found different forms of support. We will also inquire directly about the social ties they named to understand what factors contributed to success in building relationships and what happened in prospective relationships that did not materialize. Interview transcripts and ethnographic field notes will be imported into ATLAS.ti, a qualitative data management and analysis software program. Data will be coded using deductive and inductive approaches involving the use of a priori categories (deductive) and emergent categories (inductive) through line-by-line coding and constant comparative analysis. This process will involve the development of coding frameworks comprised of categories derived from the interview guides and expanded to include emic categories specific to the study. Data segments in both transcripts and field notes will be assigned to these categories, and the content of each category will be summarized and examined for inconsistent and contradictory evidence.

***Aim 4: Describe program implementation***: Key network-based program components and implementation characteristics (including barriers/facilitators) will be assessed through thematic inductive coding of in-depth exploratory interviews with mentors and staff. Data will be triangulated through similar coding of guest interviews. Key components identified in qualitative interviews will be quantitatively scored with Likert scales and analyzed via descriptive statistics. Acceptability and feasibility will be assessed using descriptive statistics of the AIM and FIM. In addition to calculating summary statistics for each item in the two 4-item measures, we will also create scales for each measure by averaging responses (values range 1 to 5.) We will also calculate within-person change over time on these same key measures for program participants to support estimation of program effectiveness and effect size and to allow estimation of parameters necessary for future intervention research.

### Pre-implementation data & methods

Baseline interview data collected in the summer of 2023 with two staff and five peer mentors were analyzed to inform our understanding of the program and refine study design/instrumentation. Open-ended interviews were conducted by three study authors (BFH, JG, and GZ) during a study team visit to the house. All individuals who served as staff and mentors at the time of this initial data collection provided informed consent and participated in the interviews. Interviews lasted between 30 and 60 min; they were audio recorded and transcribed. Interview guides focused on staff/mentor perceptions of key elements of the program, how these differ from traditional reentry programs, and implementation barriers/facilitators at program start-up. Interviews were semi-structured, with participants asked follow-up questions not listed in the guide when pertinent.

Data were manually coded by four study authors (BFH, JG, KB and DL), using both deductive and inductive approaches. Initially, an inductive approach was applied to allow for themes to emerge without use of a pre-determined framework. Once this stage of coding was completed, and there were clear overlaps of themes with existing organizational/programmatic frameworks, then a deductive approach was used to further revise and refine the themes using a Logic Model, as this framework was identified as a strong match with the emergent themes.

Logic Models have been promoted by Centers for Disease Control and Prevention (CDC) to support program evaluation (Centers for Disease Control and Prevention, 2024). They are visual depictions of program activities and intended effects, including the underlying theory of change (Kellogg Foundation, 2004). Within the Logic Model, outcomes are further organized by eco-social level (individual, interpersonal, community) to highlight how themes emerged across domains of social determinants of health. Eco-social theory describes how individuals are situated within overlapping environmental spheres (interpersonal, community, society), and how those interconnected spaces collectively determine health through social experiences (Krieger, 2001). The theory has been applied to describe social determinants of incarceration (Henry, 2020), which aligns with the goals of the program under study.

To ensure reliability, the four coders initially coded transcripts independently and then reviewed the codes in debriefing meetings as a group to analyze similarities and differences and reach interrater agreement on the codes chosen. The chosen codes were arranged into a codebook initially clustered under emergent themes. Emergent themes were then organized into the Logic Model. For each code, a description was developed, any relevant sub-codes providing greater specificity were described, and an example quote was taken from a participant transcript to demonstrate the code. Transcripts were then reanalyzed with refined codes to ensure best fit with the data.

### Pre-implementation findings

#### Participants

The seven peer mentor/staff participants designed and initially implemented the Cumberland House program. As such, none of them had the opportunity to be guests of the house, nor did they ever live there. The group came together through their own social network as local leaders in the lived experience of prison reentry. They all identified as formerly incarcerated men. The total years incarcerated per person ranged from 21 to 46, with an average of 29, and a standard deviation of 10. Age in years ranged from 45 to 62. with an average of 52, and a standard deviation of 7. The total years of related experience ranged from 1 to 12, with an average of 6, and a standard deviation of 6. Regarding educational background, 40% had associates degrees, 20% had some college, and 40% had General Educational Development tests (GEDs). Their level of involvement in the program varied from very little to extensive. The two-peer staff were involved in all aspects of program design and delivery, while the five peer mentors’ involvement ranged from having substantial input and providing material support, to no previous involvement at all; one participant reported that the study interview occurred on his first day at the program.

**Fig. 1:**
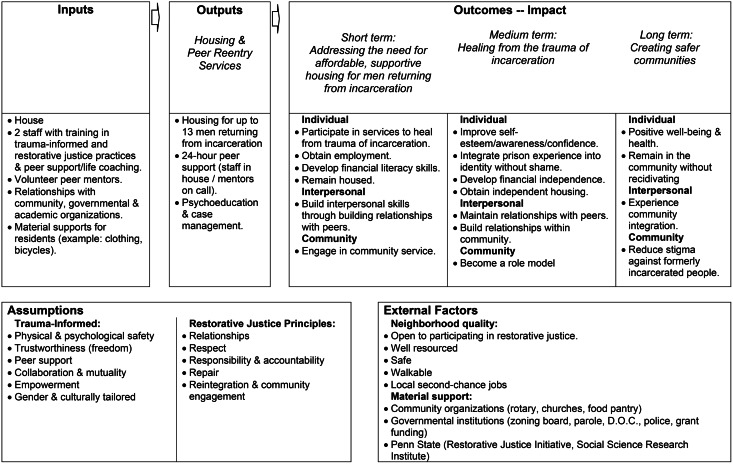
The Cumberland House Project Logic Model: Creating safer communities by addressing the need for affordable, supportive housing for men to heal from the trauma of incarceration

**Logic model**: Pre-implementation findings from thematic coding of baseline interviews identified the program elements that are presented in Fig. 1. Figure 1 organizes the themes into a Logic Model that describes how the program is expected to function. At the top we list the program’s mission: “Creating safer communities by addressing the need for affordable, supportive housing for men to heal from the trauma of incarceration.” On the left side of the model is a box containing the Inputs, or resources required for the program to function. The model flows to the right with boxes connected via arrows to show how those inputs theoretically produce outputs (program services/activities) and then eventually outcomes (expected program impacts). Outcomes are stratified across time (short, medium, long) and level of impact (individual, interpersonal, community). Outcome stratification over time is also further described with the appropriate elements from the mission (short term: addressing the need for affordable, supportive housing for men; medium term: healing from the trauma of incarceration; and long term: creating safer communities). The unconnected Assumptions box contains theoretical underpinnings that guide the model, and the unconnected External Factors box contains elements that impact the program but are outside the control of the program.

#### Inputs

Program inputs include the house and 2 paid peer staff. Staff came to the program trained in peer support, principles of trauma-informed care, and restorative justice. At the time of data collection, shortly after the creation of the program, there were 5 volunteer peer mentors. As guests move out of the house in the future, staff aims to recruit these former guests as new mentors. There are no formal qualifications to be a peer mentor aside from having a shared lived experience with the guests of the program—meaning that mentors are also formerly incarcerated men.

The program is supported by formal and informal relationships with community, governmental, and academic organizations. For example, local churches and non-profit organizations have donated material resources and provided non-material support to the start-up of the program. Examples of material resources include bicycles, and access to a food pantry. Non-material support includes voicing support for the program in public forums and facilitating what staff/mentors perceived as a welcoming neighborhood culture towards the program. Relationships with governmental entities have led to nearly all guest referrals and provided non-material support to facilitate grant applications. Most initial guest referrals came from the Department of Corrections (DOC), while the Parole Board permitted exceptions to guests to allow them to form and maintain peer contacts with other formerly incarcerated people. Academic organizations have provided training and networking opportunities which expanded staff capacity.

**Outputs**: Staff and mentors described the program as providing safe, affordable, and supportive housing for men to heal from the trauma of incarceration. The house was designed to be home-like and eliminate any elements that might replicate a carceral experience. Peer staff/mentors are on call 24 h a day, seven days a week to provide emotional support through active listening and reassurance. They also provide minimally structured case management and tailored psychoeducation. Ideally staff/mentors would provide assistance that matches the guests needs, including intensive help across the full spectrum of social needs (for example: obtaining identification, adapting to technology, navigating shopping/romantic relationships, obtaining employment, and addressing health needs). However, staff/mentor relationships with guests are supposed to develop organically and be based on individual affinity and needs. Therefore, there is no formal process of mentor assignment or oversight of mentor activities. The social ties and networks formed with peer staff/mentors are seen by the peer program staff/mentors as the primary program mechanisms underlying the expected outcomes.

#### Outcomes

Outputs in the logic model are organized across 3 levels (individual, interpersonal, and community) to highlight the building of social ties and networks, as well as other expected outcomes, across these levels. Short-term outcomes at the individual level include participating in services to address trauma from incarceration, obtaining employment, developing financial literacy skills, and remaining housed. At the interpersonal level, guests are expected to develop interpersonal skills and relationships with peers in the program. At the community level, guests will engage in community service, which the peer staff facilitate through planning voluntary house outings such as park clean-ups. Medium-term outcomes at the individual level include improving self-esteem/awareness/confidence, integrating the prison experience into identity without shame, developing financial independence, and obtaining independent housing. At the interpersonal level, participants will maintain relationships with peers and build relationships within the community. Long-term outcomes at the individual level include achieving positive well-being and health and avoiding rearrest/reincarceration to remain in the community. At the interpersonal level, participants will experience community integration. Finally, the program aims to reduce stigma against formerly incarcerated people at the community level. However, the scope of our measures will focus on perceptions of stigma by program participants.

### Assumptions

Staff/mentors reported that the underlying theory of change is based on their training in trauma-informed care and restorative justice and their own experiences underscoring the importance of mutual support during reentry. The frameworks of trauma-informed care and restorative justice center relationship building and repair as central mechanisms of healing and program effectiveness. In line with best practices of trauma-informed care, the program explicitly seeks to promote physical and psychological safety, trustworthiness, peer support, collaboration/mutuality, and empowerment, and it seeks to tailor program services to participants’ gender and culture. Physical and psychological safety are promoted through the intentional location of the program in a residential house in what staff/mentors perceived to be a “high-quality” neighborhood. The physical design of the house is also modeled to avoid references to carceral experiences; for example, participants can name their bedrooms, rather than staff assigning them numbers. The goal of this design is to avoid psychological triggers to the trauma of incarceration, to create feelings of freedom, and to model that participants are trusted. Peer support with a focus on collaboration, mutuality, and empowerment are the key principles underlying the mentoring and network building elements of the program model. The program is also specifically designed by formerly incarcerated older men for formerly incarcerated older men to attend to the unique gender, age, and cultural needs of this population.

Key elements of restorative justice practices which are central to the program model include: relationships, respect, responsibility, accountability, repair, reintegration, and community engagement. Outcomes at the interpersonal and community levels leverage theories of change related to restorative justice, where individuals engaging in relationships with the community in respectful and responsible ways can demonstrate accountability and facilitate individual repair/reintegration while also promoting broader acceptance of formerly incarcerated people within the community.

External Factors: access to a “high-quality” neighborhood (as perceived by staff/mentors) and material resources from the community and other organizations were cited as important external factors contributing to the program’s functioning. Relevant factors to neighborhood quality were described as: openness to participating in restorative justice, well-resourced, safe, walkable, and close access to second-chance jobs. Openness to participating in restorative justice was described by staff/mentors as a cultural perspective they believed was shared by many community members where the community generally did not hold stigmatizing views about people who were formerly incarcerated and/or were committed to forgiveness for this population. Well-resourced highlights the importance of available local resources, including food pantries, health/social services, parks, and transportation. Safe refers to the absence of neighborhood violence, gangs, and open drug sales/use. Walkable was cited as very important given the challenges the participants often faced obtaining driver’s licenses and personal vehicles. Local second-chance jobs related both to the openness of the community in participating in restorative justice, and the availability of jobs which could realistically employ people with serious convictions

## Discussion

The pre-implementation findings and logic model have been integrated into the research methods described above to inform ongoing updates to data collection and measures. It also provides a theory of change, as understood by the Cumberland House program developers and implementers, which serves as a reference point for our research team to compare to published literature. Results highlighted the primary intervention of peer support and revealed overlaps between the program’s theory of change and existing frameworks of trauma-informed care and restorative justice.

As initially described, the Cumberland House program’s approach is bolstered by research on the benefits of peer support for this population, with currently and formerly incarcerated individuals reporting that shared experiences of incarceration, reentry, and trauma can facilitate the building of trust with peer mentors who can then facilitate connections to valuable resources (Matthews, 2021; Reingle Gonzalez et al., 2019). Our study is poised to extend these findings by describing how such peer relationships underlie health and social outcomes.

The Cumberland House program leverages principles of trauma-informed care by integrating formally trained staff with lived experiences of incarceration to continually assess and respond to guest needs throughout the reentry process. Central to trauma-informed care is the incorporation of culturally and gender responsive services. The program addresses this by working to address stigma related to prior incarceration which can be a barrier to locating housing, securing employment, forging new social ties, and receiving adequate health care services, contributing to poor mental health outcomes (Western et al., 2015). The gender responsive element of the program is responsive to how in the criminal justice system, men are treated more harshly (Henry, 2022), have less access to rehabilitative services (Henry & Gray, 2024), and have fewer social ties than women (Western et al., 2015). The development of this program has the potential for wide impact, as there have been fewer investments in trauma-informed services for men, despite a desperate need due to high exposure to trauma and much higher rates of incarceration for men (Vaswani et al., 2021). Further research on trauma informed services is in its nascency and lacks evidence for the effectiveness of reentry services for adult men, heightening this study’s potential contributions.

The Cumberland House program also infuses principles of restorative justice, as represented in the logic model. They leverage these principles through the focus on building peer relationships and demonstrating respect through the homelike environment of the house. The expectations of participating in community service and peer mentoring provide opportunities for developing personal responsibility, interpersonal repair and community reintegration. While novel, this is not the first reentry program to incorporate principles of restorative justice. There has been recent interest in expanding the use of restorative justice into reentry programs, with some early positive results regarding potential to reduce recidivism (Skinner-Osei, & Osei, 2024). Further, structured restorative justice programs delivered during incarceration have been linked to reduced recidivism after release (Han et al., 2021; Richner et al., 2023).

While our study has many strengths, it is not without limitations. Pre-implementation analysis and findings presented here are descriptive, and participant demographics from this portion of our study are unlikely to be generalizable to future house guests or the general reentry population. It is also extremely difficult to create a comparison group for this population. While we apply all available relevant administrative data points to match participants, later findings may not be completely generalizable. Nonetheless, our study’s future analysis will describe potential outcomes and impacts of this novel community building program for older men returning from incarceration. Our innovative methodological approach will provide new information on the social network mechanisms underlying the individual change experienced by program participants.

## Data Availability

Data are currently unavailable as the study is ongoing.

## Abbreviations

AIM: Acceptability of Intervention Measure
ARPA: American Rescue Plan Act
CAPI: Computer-Assisted Personal Interview
CDC: Centers for Disease Control and Prevention
DOC: Department of Corrections
FIM: Feasibility of Intervention Measure
GED: General Educational Development tests
ERGM: Exponential Random Graph Models
MSPSS: Multidimensional Scale of Perceived Social Support
NSHAP: National Social Life, Health, and Aging Project
PHQ: Patient Health Questionnaire
SAMHSA: Substance Abuse and Mental Health Services Administration
SAOM: Stochastic Actor-Oriented Models
TAPS: Tobacco, Alcohol, Prescription medications, and other Substance
US: United States
WHOQOL: World Health Organization Quality of Life
